# A Structural Equation Model to Explain Gambling Problem Severity in Adolescents with an Integrated Perspective

**DOI:** 10.1007/s10899-023-10266-3

**Published:** 2023-11-19

**Authors:** Maria Anna Donati, Kimmo Vehkalahti, Daniela Capitanucci, Caterina Primi

**Affiliations:** 1 NEUROFARBA Department, University of Florence, Via di San Salvi 12 – Padiglione 26, 50135 Florence, Italy; 2 Centre for Social Data Science, CSDS, University of Helsinki, Helsinki, Finland; 3 Association AND [Azzardo e Nuove Dipendenze], Varese, Italy

**Keywords:** Adolescents, Gambling, Structural equation models (SEMs), Indirect Effects, Prevention

## Abstract

To be effective in the prevention of adolescent problem gambling, it is fundamental to enhance knowledge about the antecedents of gambling problem severity and the mechanisms through which these dimensions are related to problematic gambling behavior. This study aimed at testing how selected cognitive (correct knowledge of gambling and gambling-related cognitive distortions) and affective (positive economic perception of gambling and expectation and enjoyment and arousal towards gambling) variables are related to gambling frequency and gambling problem severity. Problem gambling was conceptualized as Gambling Disorder symptoms according to the last edition of the DSM. Participants were 447 Italian high school students (68% males, mean age = 16.8, SD = 0.84). Structural Equation Models (SEMs) conducted with adolescent gamblers attested two indirect effects from knowledge to problem gambling: One through gambling-related cognitive distortions and one through gambling frequency. Overall, results confirmed that adolescent problem gambling is a complex phenomenon explained by multiple and different factors. Practical implications for preventive efforts are discussed.

## Introduction

In the last years, there has been a rapid increase of the prevalence of adolescent gambling (see, for a review, Calado et al., 2017). In Europe, 11–33% of adolescents aged 15–16 years old reported gambling in the last 12 months, with 1.4% classified as problematic gamblers (ESPAD Group, 2020). Calado et al., 2017’ s review reported that 0.2–12.3% of youth meet criteria for problem gambling. According to the *Diagnostic and Statistical Manual of Mental Disorders* (DSM-5, American Psychiatric Association, 2013), Gambling Disorder (GD) is characterized by symptoms such as preoccupation with gambling, risked relationships because of gambling habits, and inability to control or stop gambling. As early gambling onset is associated with more severe gambling-related problems in adulthood (Dowling et al., 2017), prevention of gambling is an important public health issue (Messerlian et al., 2005). Accordingly, enhanced knowledge about the antecedents of gambling problem severity in adolescence is wanted to develop theory-driven preventive initiatives (Keen et al., 2017) and to change behavior (St Quinton et al., 2022).

With this consideration in mind, the current study aimed at testing how different cognitive and affective factors are related to GD symptoms among adolescents according to an integrated perspective that helps scholars in understanding the gambling phenomenon and in preventing it (e.g., Calado et al., 2020; Canale et al., 2015; Donati et al., 2013; Ladouceur, 2001). Indeed, there can be identified both cognitive and affective variables as predictors of problem gambling in adolescence. Concerning the cognitive variables, knowledge about gambling and reasoning about randomness are important negative predictors of gambling problems, i.e., adolescents more aware of the gambling nature and harms are less susceptible to cognitive distortions and false beliefs about gambling, and youth more able to reason in probabilistic terms, as well as less susceptible to probabilistic biases and heuristics, are less likely to develop gambling problems (e.g., Delfabbro et al., 2006; Delfabbro et al., 2009; Donati et al., 2013). Instead, youth with higher levels of cognitive distortions related to gambling have been found to be more prone to problem gambling as they have erroneous beliefs about the independence of random gambling events and tend to overestimate their chances of winning (e.g., Cosenza et al., 2019; Delfabbro et al., 2006; Donati et al., 2018). Regarding affective variables, adolescents characterized by positive perceptions, attitudes and outcome expectations toward gambling, are more likely to engage in gambling behavior and to be problem gamblers (e.g., Gillespie et al., 2007; Savard et al., 2018). For instance, gambling problems in adolescents are predicted by the perception of gambling as a profitable economic activity (e.g., Delfabbro et al., 2006; Delfabbro et al., 2009; Donati et al., 2013) and by positive attitudes toward gambling in terms of harmfulness (e.g., Derevensky et al., 2010; Monaghan et al., 2008). The expectancy that gambling is a way to reach positive emotions and outcomes (e.g., Canale et al., 2015; Donati et al., 2022b; Huic et al., 2017; Wickwire et al., 2010) is also at the basis of gambling behavior.

Following these considerations, we aimed to develop and test an integrated model in which both cognitive and affective variables are taken into account to explain GD symptoms in adolescents. In fact, despite the above-described bivariate relationships among those variables and gambling behavior, to date no study has investigated the interrelation between the mentioned dimensions in a comprehensive framework. Instead, the description of the relations between these dimensions would be crucial to understand how they mediate and affect each other, and eventually are associated with GD symptoms, outlining opportunities for a multidimensional intervention and more effective prevention policies.

**Fig. 1:**
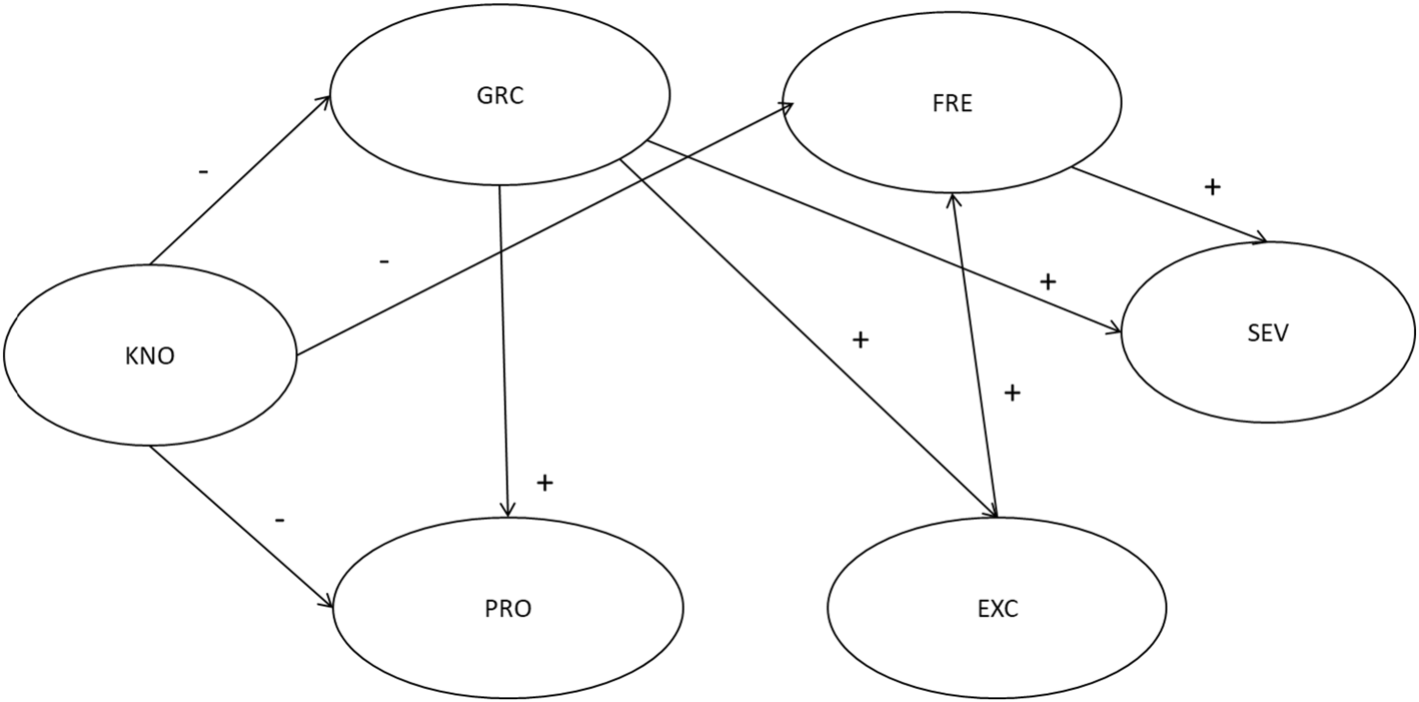
Hypothesized direct paths among the variables. *KNO* Correct knowledge about gambling, *PRO* Positive economic perception of gambling,* GRC* Gambling-related cognitive distortions,* EXC* Expectation and enjoyment and arousal towards gambling,* FRE* Gambling frequency,* SEV* Gambling Disorder

Based on the previously found bivariate relationships, our integrated model predicted that *correct knowledge of gambling* (KNO) would be the independent variable. Knowledge was hypothesized to be negatively related to the *positive economic perception of gambling* (PRO) (Delfabbro et al., 2009), to *gambling-related cognitive distortions* (GRC) (Ladouceur et al., 2004), and to gambling frequency (FRE) (Donati et al., 2019). In turn, erroneous beliefs about gambling were thought to be positively related to *positive economic perception of gambling* (PRO) (Delfabbro et al., 2009), to the *expectation and enjoyment and arousal towards gambling* (EXC) (Gillespie et al., 2007), and to GD symptoms (SEV) (Donati et al., 2018). We hypothesized that expectation related to gambling concerning the enjoyment and arousal domain would be the antecedents of gambling frequency (Gillespie et al., 2007; Wickwire et al., 2010), which, in turn, would be positively associated with GD symptoms (Vachon et al., 2004). In addition to the direct effects, we were interested in analyzing the indirect effects, i.e., the effects of correct knowledge of gambling on GD symptoms through the mediational role of the positive economic perception of gambling, gambling-related cognitive distortions, expectation of enjoyment and arousal towards gambling, and gambling frequency (see Fig. 1 for the representation of the hypothesized model).

To test the hypothesized model, we used Structural Equation Models (SEMs). Most of the studies carried out so far are based on bivariate linear perspectives that have not considered the simultaneous effects of different factors on gambling-related negative consequences. Indeed, only in the minority of the cases, statistical approaches as SEMs have been applied (e.g., Calado et al., 2020; Leon‑Jariego et al., 2020). Thus, knowledge about the underlying mechanisms through which various risk/protective factors are simultaneously related to problem gambling on the light of theoretical perspective is still poor. Instead, the use of SEMs to test theory-based mediational questions has been suggested and recommended as a flexible and powerful statistical mean for testing hypotheses in various research domains, particularly in the field of risky behaviors (Bryan et al., 2007).

## Methods

### Participants

Participants were 447 students (68% males) between the ages of 15 and 20 years (mean age = 16.8, *SD* = 0.84). The sample was recruited from different types of high schools in urban centers of the north of Italy. The study procedures were carried out in accordance with the Declaration of Helsinki. The detailed study protocol explaining the research aim and methodology was approved by the institutional review board of each school. The students received an information sheet on the study; it assured them that the data obtained would be handled confidentially and anonymously.

They also were asked to provide written informed consent. Parents of minors were required to provide consent on behalf of their children.

### Measures and Procedure

To investigate gambling frequency and GD symptoms, we administered the *Gambling Behavior Scale for Adolescents* (GBS-A; Primi et al., 2015), composed of two sections. The first one consists of unscored items investigating gambling frequency through ten items assessing the frequency of participation (never, sometimes in the year, sometimes in the month, sometimes in the week, daily) during the last year in ten gambling activities (card games, bets on games of personal skill, bets on sports games, bets on horse races, bingo, slot machines, scratch cards, lotteries, online games, and private bets with friends). Based on their responses to this section, participants can be classified as *non-gamblers* (no gambling behavior) and *gamblers* (gambling on at least one activity; Welte et al., 2009). Among gamblers, *non-regular gamblers* (i.e., those who participated from less than monthly to less than weekly in at least one gambling activity) and *regular gamblers* (i.e., those who participated weekly or daily in at least one gambling activity) can be identified (Winters et al., 1993).

The second section is composed of nine scored items assessing the DSM-5 diagnostic criteria of GD. Each item is evaluated on a 3-point Likert scale ranging from 0 (*Never*) to 2 (*Often*). An example of item is “*Have you gambled more money than you could allow?*”. Based on the responses to this section, for each respondent is possible to derive an Item Response Theory (IRT)- based score.

To measure gambling-related knowledge, the *Gambling Related Knowledge Scale-For Adolescents* (GRKS-A; Donati et al., 2019) was employed. This is a short self-report scale aimed at assessing adolescents’ individual knowledge about gambling relative to its nature, functioning, and risks. It is composed of eight items with a 4-point Likert scale (from 1 = *totally disagree*, to 4 = *totally agree*). An example of item is “*In gambling, small winnings stimulate people to gambling again*”.

The *Gambling Related Cognitions Scale-Revised for Adolescents* (GRCS-RA; Donati et al., 2022a), derived from the original GRCS (Raylu & Oei, 2004), was used to assess gambling-related cognitive distortions. The GRCS-RA contains 14 Likert-type items with a 5-point scale ranging from 1 (*strongly disagree*) to 5 (*strongly agree*), measuring the tree specific gambling-related biases, i.e., *Illusion of Control* (4 items), *Predictive Control* (6 items), and *Interpretative Bias* (4 items). An example of item is “*Specific numbers and colors can help increase the chances of winning in gambling*” (*Illusion of Control*).

For the perception of economic profitability of gambling, the *Profitability* subscale of the *Gambling Attitude Scale* (GAS, Delfabbro & Thrupp, 2003; Italian version: Primi et al., 2013) was used. It contains 4 Likert-type items, with a 5-point scale ranging from *strongly disagree* to *strongly agree*. An example of an item is “*You can make a living from gambling*”.

The *Enjoyment/Arousal* subscale of the *Gambling Expectancies Questionnaire*-*Modified* (GEQ; Gillespie et al., 2007; GEQ—Donati et al., 2022b) was employed to assess expected arousal-related benefits about gambling related to enjoyment, excitement, relief from boredom, escape/tension reduction, and social interaction. The subscale is composed of seven Likert-type items ranging from 1 = *strongly disagree* to 5 = *strongly agree*. An example of an item is “*Feeling more relaxed*”.

### Statistical Analyses

We used IBM SPSS Statistics 24.0 (IBM Corp., Armonk, NY, USA) to conduct the descriptive analysis, and the statistical programming language R (R Core Team, 2022) with the RStudio software (RStudio, PBC, Boston, MA, USA) to conduct the structural equation modelling, including the serial mediation models (Hayes, 2017; Hayes & Preacher, 2013). All structural equation models were estimated using the *lavaan* package of R (Rosseel, 2012). A robust variant of the Maximum Likelihood estimator (“MLM”) was employed in the model fitting. Throughout the modelling, *p* values < 0.05 were considered statistically significant. The path model with the estimated coefficients was drawn using the semPlot package of R (Epskamp, 2019).

To decrease the number of the items and to increase their measurement reliability, we created 18 item parcels from the 52 original items, by systematically averaging the best and worst items based on their factor loadings (Little, 2013). Each item parcel was formed from two to four items.

With regard to the fit measures, we reported the robust versions of the following fit indices: the Standardized Root Mean square Residual (SRMR, Jöreskog & Sörbom, 1993), the Root Mean Square Error of Approximation (RMSEA,Steiger & Lind, 1980), and the Comparative Fit Index (CFI, Bentler, 1990). Ideally, for a model that fits the data, the SRMR would be close to 0.08 or lower, the CFI would be 0.95 or higher (Hu & Bentler, 1999) and the RMSEA values would be less than 0.08 (Steiger & Lind, 1980).

## Results

Prior to conducting the analyses, we looked at missing values in the data. Starting from the assumption that missing values for gambling frequency and GD symptoms could not be replaced by a missing data treatment, a listwise deletion was conducted excluding cases for which these two scores were missing, i.e., those participants who did not respond to one or more items at the GBS-A section A and B. Only 1.4% (*n* = 6) of participants did not respond to the Section A assessing gambling frequency, and 4.5% (*n* = 20) of the sample missed to respond to the Section B relative to GD symptoms. Thus, the dataset comprised 421 participants.

### Gambling Behavior Description

The results indicated that 110 adolescents were non-gamblers (26%). Thus, further analyses were conducted only with the gamblers (*n* = 311). Among them, 24% were regular gamblers. The most common activities were scratch-tickets (68%), bingo (52%) and cards for money (48%). Adolescents gambled predominantly with friends (75%) and familiars (75%), with 25% of the sample who declared to gamble alone or with the partner (13%).

### Gambling Modeling

Table 1 displays the latent factors, their short labels, and the number of the original items and item parcels measuring them.

**Table 1:** Latent factors, their labels, number of original items, item parcels, and sources

Latent factor	Items	Parcels	Label	Source
Gambling severity (SEV)	9	3	SevP1–SevP2–SevP3	GBS-A (Primi et al., 2015)
Gambling frequency (FRE)	10	3	FreP1–FreP2–FreP3	GBS-A (Primi et al., 2015)
Gambling-related knowledge (KNO)	8	3	KnoP1–KnoP2–KnoP3	GRKS-A (Donati et al., 2019)
Gambling-related cognitive distortions (GRC)	14	4	GrcP1–GrcP2–GrcP3–GrcP4	GRCS-RA (Donati et al., 2022a)
Perception of gambling profitability (PRO)	4	2	ProP1–ProP2	GAS (Delfabbro & Thrupp, 2003; Italian version: Primi et al., 2013)
Expected arousal-related benefits about gambling (EXC)	7	3	ExcP1–ExcP2–ExcP3	GEQ-MOD (Donati et al., 2022b)

* GBS-A* Gambling Behavior Scale-for Adolescents,* GRKS-A* Gambling-Related Knowledge Scale-Adolescents,* GRCS-RA* Gambling-Related Cognitions Scale-Revised for Adolescents,* GAS* Gambling Attitude Scale,* GEQ-MOD* Gambling Expectancies Questionnaire-Modified

Preliminarily, we computed the bivariate correlations among the item parcels (Table 2).

**Table 2:** Bivariate correlations among the item parcels

	KnoP1	KnoP2	KnoP3	ProP1	ProP2	GrcP1	GrcP2	GrcP3	GrcP4	ExcP1	ExcP2	ExcP3	FreP1	FreP2	FreP3	SevP1	SevP2
KnoP2	0.53***																
KnoP3	0.43***	0.47***															
ProP1	− 0.23***	− 0.27***	− 0.27***														
ProP2	− 0.23***	− 0.24***	− 0.26***	0.59***													
GrcP1	− 0.17**	− 0.21***	− 0.22***	0.32***	0.35***												
GrcP2	− 0.26***	− 0.18**	− 0.21***	0.33***	0.33***	0.68***											
GrcP3	− 0.18**	− 0.15**	− 0.18**	0.27***	0.24***	0.62***	0.68***										
GrcP4	− 0.12*	− 0.09	− 0.12*	0.29***	0.27***	0.66***	0.69***	0.64***									
ExcP1	− 0.13*	− 0.10	− 0.20***	0.12*	0.21***	0.27***	0.27***	0.23***	0.28***								
ExcP2	− 0.05	− 0.04	− 0.18**	0.14*	0.18**	0.20***	0.17**	0.17**	0.23***	0.70***							
ExcP3	− 0.03	− 0.04	− 0.18**	0.14*	0.17**	0.19**	0.18**	0.14*	0.20***	0.68***	0.66***						
FreP1	− 0.21***	− 0.31***	− 0.24***	0.30***	0.28***	0.26***	0.27***	0.22***	0.26***	0.19***	0.18**	0.14*					
FreP2	− 0.14*	− 0.23***	− 0.13*	0.16**	0.11*	0.14*	0.16**	0.14*	0.19***	0.26***	0.22***	0.21***	0.46***				
FreP3	− 0.11	− 0.16**	− 0.16**	0.20***	0.19***	0.15**	0.20***	0.21***	0.19***	0.24***	0.17**	0.21***	0.47***	0.44***			
SevP1	− 0.05	− 0.09	− 0.04	0.19***	0.16**	0.24***	0.27***	0.27***	0.30***	0.14*	0.14*	0.17**	0.36***	0.23***	0.20***		
SevP2	− 0.04	− 0.17**	− 0.09	0.17**	0.12*	0.25***	0.24***	0.22***	0.26***	0.12*	0.11	0.13*	0.43***	0.30***	0.30***	0.62***	
SevP3	0.01	− 0.04	− 0.01	0.13*	0.10	0.31***	0.22***	0.28***	0.29***	0.19***	0.15**	0.15**	0.35***	0.34***	0.29***	0.52***	0.52***

*** *p* < 0.001, **p < 0.01, *p < 0.05

Then, we used the structural equation model to test the serial multiple mediation hypothesis between gambling-related knowledge (KNO) and gambling severity (SEV), through four mediators (GRC: gambling-related cognitive distortions, PRO: the perception of gambling profitability, EXC: expected arousal-related benefits about gambling, and FRE: gambling frequency). Hence, the analysis included both the direct effect from gambling-related knowledge to gambling severity and fifteen different indirect effects through the four other factors that were acting as mediators (Hayes & Preacher, 2013; Hayes, 2017). The model showed an excellent fit with a non-significant chi-square (*χ*^*2*^ = 120.8, *df* = 120, *p* = .462) and the fit indices (CFI = 0.991, RMSEA = 0.005, 90% CI [0.000, 0.031], SRMR = 0.038).

**Fig. 2:**
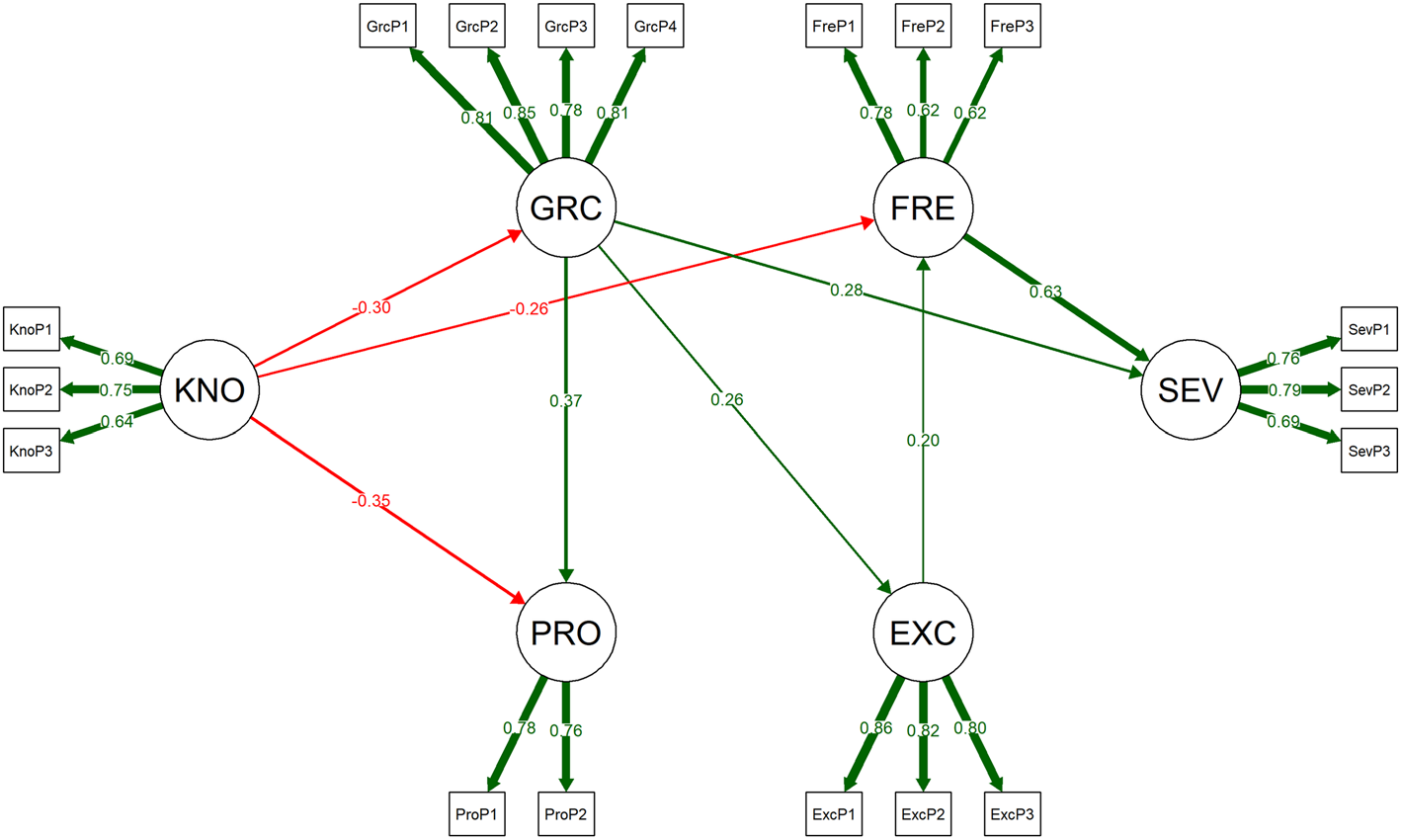
Factor loadings of the measurement models where each latent factor is measured by the corresponding item parcels, as well as the standardized estimates of the significant coefficients of the paths between the six factors in the four-mediator model. * KNO* Correct knowledge about gambling,* PRO* Positive economic perception of gambling,* GRC* Gambling-related cognitive distortions,* EXC* Expectation and enjoyment and arousal towards gambling,* FRE* Gambling frequency,* SEV* Gambling Disorder. Observed variables are the item parcels (see Table 1)

Figure 2 displays the factor loadings of the measurement models where each latent factor is measured by the corresponding item parcels, as well as the standardized estimates of the significant coefficients of the paths between the six factors in the four-mediator model. The following two paths (indirect effects with one mediator) appeared statistically significant: (1) KNO → GRC → SEV (− 0.305 × 0.282 = − 0.086), and (2) KNO → FRE → SEV (− 0.261 × 0.633 = − 0.165). As all the other indirect effects and the direct effect were non-significant, the effect between gambling-related knowledge and gambling severity was fully-mediated through those two specific paths. The sign of the product of the coefficients was negative, reflecting the fact that the gambling-related knowledge has a negative effect to the mediators while the significant paths from the mediators to gambling severity are positive.

## Discussion

To implement effective preventive interventions, it is fundamental to refer to coherent theoretically and empirically evaluated frameworks describing and explaining the antecedents of the targeted outcomes, and how they may be related to these outcomes (Flay et al., 2005; Keen et al., 2017). In this way, prevention programs should have clearer concept of the expected causal mechanisms by which the programs would exert their effect. In the research field of adolescent gambling, over the years, there has been an increasing commitment and verification of empirical models supported by theoretical foundations (e.g., Calado et al., 2020; Donati et al., 2018; Leon‑Jariego et al., 2020; Ricijas et al., 2016; St-Pierre et al., 2015). However, among these studies, there has been a relatively modest use of SEMs, that, instead, allow researchers to empirically assess theoretical models for observed and latent variables. Thus, SEMs allow to simultaneously analyze the efficacy of the measurement model—the relationships between the observed variables and the latent variables—as well as the structural model, i.e., the adequacy of the predicted relationships among the latent variables and their relative roles as exogenous and endogenous variables.

The aim of this study was to test how different cognitive and affective factors influence GD symptoms among adolescents, by applying SEMs. We confirmed that adolescent problem gambling is a complex phenomenon explained by multiple and different factors dealing with the cognitive area—knowledge of gambling and cognitive distortions about gambling, and the affective area -perception of gambling profitability and expectation of enjoyment from gambling. Two indirect effects from knowledge to GD symptoms have been found: one through gambling-related cognitive distortions, and one through gambling frequency.

Practical implications can be derived in designing preventive interventions with adolescents. As gambling-related correct knowledge has direct negative effects on erroneous beliefs about gambling, that, in turn, represents the core variable through which gambling-related correct knowledge exercises a significant indirect effect on gambling severity, preventive interventions with youth should focus on enhancing students’ knowledge. In this regard, the Keen and colleagues’ (2017) systematic review about gambling prevention interventions for adolescents attest that the studies that conducted an evaluation of the intervention’s effects were effective in improving knowledge, while it was harder to modify affective factors, and self-reported gambling behavior. Indeed, according to the dual-system model (Casey et al., 2016), there is a typical dual-characterization of the adolescent brain, with a less developed cognitive system and a particularly active socio-affective system, that makes them vulnerable to do risky decisions. This specificity of adolescence requires that, together with cognitive factors, educational efforts should be directed also to affective factors. In particular, it is important to make adolescents aware of the interaction among these factors and the risk fort a shift from a correct reasoning to heuristic and risky decisions when youth are in hot contexts, i.e., situations in which socio-emotional features as peer pressure or the possibility to gain money, that function as activators of the socio-affective system (Casey et al., 2016; Shulman et al., 2016).

The particular strength of this study is the application of SEMs to verify the adequacy of the hypothesized model, integrating the cognitive and affective dimensions, as it is previously unexplored in explaining gambling behaviour in adolescents. However, it is not without limitations. For instance, self-report data have been collected, with a risk for biases such as social desirability and memory recall. Secondly, the study employed a cross-sectional design, and therefore possible causal relationships between the variables examined cannot be inferred. Future studies are needed to confirm the model and also to verify its generalizability with adolescents of other countries.

Moreover, it has been recently suggested that false beliefs about gambling might be the precursor of problematic loot box use among undergraduate students and adults (Brooks & Clark, 2019) but also, given the growing prevalence of loot boxes, with 58% of the top mobile and desktop games on the Google Play store containing them (Zendle et al., 2020), false beliefs could be gradually built up by playing video games, long before young people get close to real gambling. In fact, buying loot boxes is linked to problem gambling and problem video gaming (Li et al., 2019 and, according with King and colleagues (2019), in-game purchasing might contribute to higher risk consumer behavior, due to some design strategies. Future studies should be realized to understand the pathway though which erroneous interpretations of gambling outcomes may be linked also to other behavioral problems in youth.

## Data Availability

The dataset analysed during the current study are available from the corresponding author on reasonable request.
